# Enhanced Stimuli-Responsive Electrorheological Property of Poly(ionic liquid)s-Capsulated Polyaniline Particles

**DOI:** 10.3390/polym9090385

**Published:** 2017-08-23

**Authors:** Chen Zheng, Yuezhen Dong, Yang Liu, Xiaopeng Zhao, Jianbo Yin

**Affiliations:** Smart Materials Laboratory, Department of Applied Physics, Northwestern Polytechnical University, Xi’an 710129, China; zhengchen2603@163.com (C.Z.); dongyuezhen@mail.nwpu.edu.cn (Y.D.); daguoyliu@mail.nwpu.edu.cn (Y.L.); xpzhao@nwpu.edu.cn (X.Z.)

**Keywords:** poly(ionic liquid)s, polyaniline, composite particles, electrorheological effect

## Abstract

We used inherently conducting polyaniline as a core to develop a type of poly(ionic liquid)s-capsulated polyaniline composite particles in order to both overcome the surface charged character of pure poly(ionic liquid)s particles prepared by post ion-exchange procedure, and enhance electrorheological (ER) effect. The structure was characterized by different techniques and the electrorheological suspension was prepared by dispersing the composite particles in silicone oil. Under electric fields, the electrorheological properties of the suspensions of poly(ionic liquid)s-capsulated polyaniline composite particles were measured and compared with their single forms. It is demonstrated that the composite particles have distinctly enhanced electrorheological effect compared with the pure poly(ionic liquid)s and polyaniline particles under electric stimuli. At 4 kV/mm of electric field, the yield stress of the suspension of poly(ionic liquid)s-capsulated polyaniline composite particles in silicone oil is about 2.3 kPa, which is twice as high as 1.2 kPa stress of the suspension of poly(ionic liquid) particles and 2.5 times as high as 0.9 kPa stress of the suspension of polyaniline particles. By using dielectric spectroscopy, microscopic observation, and oscillation rheology, we studied the origin of this enhanced electrorheological effect. The results indicated that wrapping polyaniline into poly(ionic liquid)s could partly suppress the positively charged surface state of poly(ionic liquid)s particles prepared by post ion-exchange procedure and improve the column-like electrorheological structure. This suppression should be responsible for the enhanced electrorheological effect of poly(ionic liquid)s-capsulated polyaniline composite particles.

## 1. Introduction

Smart materials have attracted much attention because they can produce an adaptive response to external stimuli [1,2]. Electrorheological (ER) suspension, which is composed of polarizable particles with a high relative dielectric constant, and an insulating liquid medium with a low relative dielectric constant, is one of the most important electroresponsive smart materials [3]. Under external electric fields, ER suspension can rapidly transfer from a low viscous Newtonian fluid into a thickening Bingham fluid due to the electrostatic interactions of polarized particles [4]. Especially, the viscosity change or phase transition is reversible and requires low energy consumption. Therefore, ER suspension has attracted significant attention as electrical–mechanical interfaces in many potential technical applications, such as automotive, aerospace, food processing, robotic, and so on [5,6,7].

To promote the real utilization of ER technology, intensive research on the development of new effective ER materials has been continuously done since Winslow first discovered ER behavior [8]. Many kinds of ER materials, such as inorganic ion compounds, inorganic semiconductors, natural organic compounds, semiconducting polymers, polyelectrolytes, carbonaceous matter, and composites, have been developed. These materials can be classified into two types of different formations, i.e., extrinsic and intrinsic ER systems [9]. The extrinsic ER system is also called a water-containing system, and includes silica gel, starch, zeolite, recent nanoparticle-based giant ER material, and so on. This system needs to adsorb a small amount of water or other polar liquids to activate the ER effect [10,11]. The function of adsorbed liquid is considered to be promoting charge carrier mobility for particle polarization, or to build a “water-bridge” between particles to induce ER effect [12]. However, problems caused by adsorbed water, such as large current density, a narrow working temperature region, and device corrosion, largely limit the application of extrinsic ER systems. Unlike the extrinsic system, the intrinsic ER system can show high ER effect in dry state; it is also called a water-free ER system. The inherently polarizable inorganics (e.g., rutile and perovskite) and some doped inorganics (e.g., zirconium-doped aluminosilicates and rare earth ion doped titania) belong to a typical intrinsic ER system, and they can show high ER effect under AC (alternating current) or DC (direct current) electric fields [13,14]. However, the practical application of the inorganic ER materials is limited by some shortcomings, such as large particle sedimentation and abrasiveness to devices. 

Compared to the inorganics, organic polymer ER materials have been considered to be more promising in real applications because of their low density, soft texture, and relatively high ER activity [15,16,17,18]. Among various organics, polyelectrolyte is most frequency studied due to its low cost, facile preparation, and high ER effect. Some classic polyelectrolytes, such as poly(sodium styrene sulfonate), poly(lithium methacrylate), and so on [19] have been used as the dispersed phase of ER suspension. However, they usually need to absorb a small amount of water or moisture to activate ER effect. In dry state, the counterions in the classic polyelectrolytes are strongly bound by the sulfonate or carboxylate groups attached to the polymer backbone. Therefore, the classic polyelectrolyte materials belong to extrinsic ER systems, and are inevitably subjected to the electrical and thermal problems mentioned above. To overcome these problems, a novel anhydrous polyelectrolyte-based ER system based on poly(ionic liquid)s (PILs) has been developed recently [20]. Different from classic polyelectrolytes, the PILs are hydrophobic due to the presence of fluorinated counterions such as bis(trifluoromethylsulfonyl)imide ((CF_3_SO_2_)_2_N^−^) and hexafluorophosphates (PF_6_^−^) [21]. Thus, the ion motion and conductivity of the PILs are without affinity to moisture or water, and as a result, the ER fluid of PIL particles exhibits ER effect in the absence of any activators [22]. Two methods can prepare hydrophobic PIL particles: the direct polymerization of hydrophobic IL monomers by different techniques, and the polymerization of hydrophilic IL monomers followed by post ion-exchange treatment [21,23,24]. When compared with direct polymerization, the post ion-exchange procedure is easier and produces a higher yield for industrial mass production. However, it is also found that PIL particles prepared by post ion-exchange procedure are easily surface charged, which sometimes results in the unexpected dependence of ER effect on electrode polarity. In addition, it is also necessary for PILs to improve ER effect for practical applications.

In this paper, we used inherently conducting polyaniline as a core to develop a type of poly(ionic liquid)s-capsulated polyaniline (PIL-c-PANI) composite particles in order to overcome the surface charged character of pure PIL particles prepared by post ion-exchange procedure and enhance ER effect. To form the capsulated composite, an aqueous dispersion was firstly prepared by polymerizing aniline in the presence of poly(*p*-vinylbenzyl trimethylammonium chloride) as steric stabilizer. By the addition of hexafluorophosphate potassium salt, the polymeric stabilizer became hydrophobic, precipitating in water and trapping polyaniline inside poly(*p*-vinylbenzyl trimethylammonium hexafluorophosphate) to form PIL-c-PANI composite particles. The ER suspension was prepared by dispersing the composite particles in silicone oil. Under electric fields, we measured and compared the ER effect of the PIL-c-PANI particles with their single forms. It was found that the PIL-c-PANI particles have a distinct enhancement in ER effect when compared with that of pure PIL particles and PANI particles, which is twice as high as the suspension of PIL particles and 2.5 times as high as the suspension of PANI particles. By using dielectric spectroscopy, microscopic observation, and oscillation rheology, we studied the origin of enhanced electrorheological effect. The results indicated that wrapping PANI into PILs could suppress the positively charged nature of present polycation-type PIL particles and improve the column-like ER structure, and this should be responsible for the enhanced ER effect of PIL-c-PANI particles.

## 2. Materials and Methods

### 2.1. Materials

2,2′-azobis(isoheptonitrile) (AIBN, Sinopharm Chemical Reagent Co., Ltd., Shanghai, China) was purified by recrystallization in ethanol. (Vinylbenzyl)trimethylammonium chloride ([VBTMA]Cl, 99%, Sigma-Aldrich Corp., St. Louis, MO, USA), potassium hexafluorophosphate (K[PF_6_], 99%, J&K Chemical, Beijing, China), aniline (Sinopharm Chemical Reagent Co., Ltd., Shanghai, China), and ammonium persulfate (APS, Tianli Chemical Reagent Co., Ltd., Tianjin, China) were used as received.

### 2.2. Preparation of PIL-c-PANI Particles

PIL-c-PANI particles were prepared by the method of Rebeca Marcilla et al. with a slight modification [25]. First, poly(vinylbenzyl)trimethylammonium chloride (P[VBTMA]Cl) was prepared by radical polymerization of 2.00 g [VBTMA]Cl in 20 mL ethanol with 0.03 g AIBN as initiator under nitrogen atmosphere. The reaction temperature was 70 °C. After reaction for 12 h, a large amount of acetone was added into this mixture to form precipitate. The precipitate was washed with acetone and vacuum dried to obtain white P[VBTMA]Cl solid. Then, 1.00 g P[VBTMA]Cl and 0.10 g aniline were dissolved in 30 mL distilled water. We added 0.25 g APS dissolved in 10 mL water dropwise into the P[VBTMA]Cl/aniline solution. After reaction for 24 h at 10 °C, a dark green PANI/P[VBTMA]Cl aqueous dispersion was obtained. Finally, an aqueous solution containing 2.00 g K[PF_6_] was poured into the PANI/P[VBTMA]Cl aqueous dispersion for ion exchange. Meanwhile, dark green gelatinous precipitate was formed. The precipitate was washed with deionized water and further soaked in 60 mL of NH_3_·H_2_O (3 wt %) for 5 min to obtain P[VBTMA][PF_6_]-capsulated PANI (P[VBTMA][PF_6_]-c-PANI) composite particles.

Pure P[VBTMA][PF_6_] particles were prepared by an ion exchange reaction of P[VBTMA]Cl with K[PF_6_] aqueous solution. Simply, 2.00 g [VBTMA]Cl was dissolved in 20 mL ethanol and then 0.03 g AIBN was added. After reaction for 12 h at 70 °C under nitrogen atmosphere, a large amount of acetone was added into this mixture to obtain white P[VBTMA]Cl solid precipitate. Then, the P[VBTMA]Cl was dissolved into water and mixed with an aqueous solution containing 2.00 g K[PF_6_] to obtain final P[VBTMA][PF_6_] particles. 

Pure PANI particles were prepared as follows: 1.00 g aniline was dissolved in 30 mL distilled water, and then an aqueous solution containing 2.50 g APS in 10 mL water was added dropwise into the aniline solution. After reaction for 24 h at 10 °C under stirring, a dark green PANI precipitate was obtained. The precipitate was washed with DI water and further soaked in 60 mL of NH_3_·H_2_O (3 wt %) for 5 min to obtain the final PANI particles.

### 2.3. Preparation of ER Suspensions

The P[VBTMA][PF_6_], P[VBTMA][PF_6_]-c-PANI, and PANI particles were further washed with water and ethanol, and dried in a vacuum at 100 °C for 48 h. The dry particles were mixed into dimethyl silicone oil (KF-96, Shin-Etsu Chemical Co., Ltd., Tokyo, Japan,) with a kinetic viscosity of 50 cSt at 25 °C with a particle concentration of 27 vol %. The density of particles was measured by a pycnometer method.

### 2.4. Characterization and Measurements

The morphology and structure of samples were characterized by different techniques. Optical microscopy (OM, Nikon ALPHAPHOT-2, Tokyo, Japan) and scanning electron microscopy (SEM, Hitachi TM3000, Tokyo, Japan) were used to observe the morphology of samples. Fourier transform-infrared spectrum (FT-IR, JASCO FT/IR-470 Plus, Tokyo, Japan) was used to analyze the chemical groups of samples. A thermogravimetric analyzer (TGA, Netzsch STA449F3, Selb, Germany) test was conducted with a heating rate of 20 °C min^−1^ within 30–800 °C in flow air to determine the thermal decomposition. A particle analyzer (Malvern Zetasizer Nano, Malvern, UK) was used to measure the Zeta potentials of samples. In the measurement, the particles were dispersed in ethanol to form dilute solution.

The ER effect of suspensions was measured by a stress-controlled rheometer (Thermal-Haake RS600, Karlsruhe, Germany) with a parallel plate measurement system at room temperature. The diameter of the plate was 35 mm, and the gap between two plates was 1.0 mm. A DC high-voltage generator (WYZ-010, Beijing, China) applied the electric field through the two plates. The flow curves of the shear stress as a function of shear rate were measured by the controlled shear rate mode in the range of 0.1–1000 s^−1^. In the dynamic oscillatory test, the modulus curves of storage modulus (*G*′) & loss modulus (*G*″) as a function of shear stress amplitude were obtained by the controlled stress mode at a constant oscillation frequency of 1.0 Hz. After one test, we exchanged the polarity of the rotation side of an electrode: the previous positive electrode would become a negative electrode at the next test, and this cycle repeated. Before every test started, the suspensions were pre-sheared for 60 s at 300 s^−1^ to remove structure history, and then the electric field was applied for 30 s in order to form an equilibrium gap-spanning ER structure.

The conductivity (*σ_p_*) of P[VBTMA][PF_6_], P[VBTMA][PF_6_]-c-PANI, and PANI particles was measured by suspension method. In this method, the leaking current density (*j*) through suspension under the electric field (*E*) s was firstly detected by galvanometer, and then the conductivity (*σ*) of suspension was calculated by *σ = j*/*E*. Because the structure form in ER suspensions under a high electric field is similar to that of anisotropic fibrous composite, *σ_p_* can be calculated by the approximate mixture equation *σ* = *φσ_p_* + (1 − *φ*)*σ_f_* (where *σ_f_* is the conductivity of silicone oil, and *φ* is the volume fraction of particles in suspension.).

The dielectric property of suspensions was measured at room temperature by an impedance analyzer (HP 4284A, Santa Clara, CA, USA), with a liquid measuring fixture (HP 16452A, Santa Clara, CA, USA) in the frequency range of 20–10^6^ Hz. The bias electrical potential was 1 V, which could not induce fibrous-like structure formation; thus, we could well compare the different polarization characteristics of different samples. The dielectric characteristic was obtained by the Cole-Cole function described as Dong et al. [22].

The ER structure formed between two electrodes was observed by an optical microscope (Nikon ALPHAPHOT-2). The gap of electrodes was 1.0 mm. Before microscopic observation, 2 kV/m of electric field was applied to the suspension in the gap for 30 s in order to form an equilibrium gap-spanning ER structure.

## 3. Results

### 3.1. Synthesis of PIL-c-PANI Particles

**Scheme 1:**
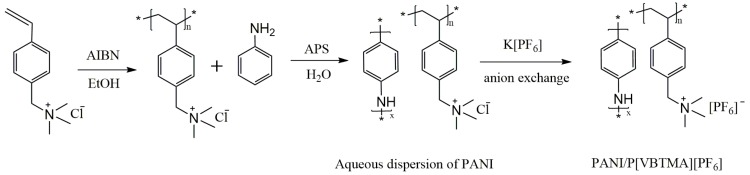
Preparation process of poly(vinylbenzyl)trimethylammonium hexafluorophosphate-capsulated polyaniline (P[VBTMA][PF_6_]-c-PANI) particles.

The preparation process of P[VBTMA][PF_6_]-c-PANI particles is illustrated in Scheme 1. First, the [VBTMA]Cl monomer was polymerized in ethanol by solution polymerization, and the product of P[VBTMA]Cl was precipitated by pouring a lot of acetone into solution. The solid polymer of P[VBTMA]Cl could be obtained after washing and evaporation drying. It was worth mentioning that the solid polymer was soluble in water. Second, PANI was synthesized by chemical oxidative polymerization in an aqueous solution of P[VBTMA]Cl as stabilizer. The perfectly clear solution became a dark-green aqueous dispersion with no precipitate appearing after reaction. Third, K[PF_6_] aqueous solution was added into the aqueous dispersion containing P[VBTMA]Cl-stabilized PANI particles. Meanwhile, an anion exchange reaction took place to replace Cl^−^ by PF_6_^−^. As a result, the polymeric stabilizer P[VBTMA]Cl became hydrophobic P[VBTMA][PF_6_], precipitating in water and trapping PANI inside to form P[VBTMA][PF_6_]-capsulated PANI composite particles.

### 3.2. Characterization of PIL-c-PANI Particles

#### 3.2.1. Morphology and Structure Analysis

**Figure 1:**
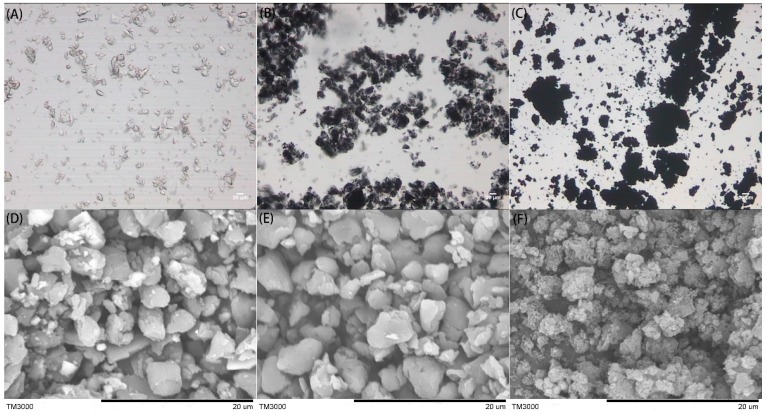
Optical microscopy photos (**A**–**C**) and scanning electron microscopy (SEM) images (**D**–**F**) of samples: (**A**,**D**) P[VBTMA][PF_6_]; (**B**,**E**) P[VBTMA][PF_6_]-c-PANI(40 wt %); (**C**,**F**) polyaniline (PANI).

Figure 1A–C shows the optical microscopy photo of P[VBTMA][PF_6_], P[VBTMA][PF_6_]-c-PANI, and PANI particles. It can be seen that P[VBTMA][PF_6_] particles are transparent and PANI particles are dark, while P[VBTMA][PF_6_]-c-PANI are dark particles encapsulated by a transparent shell. More detailed morphology can be observed by SEM images. As shown in Figure 1D,F, P[VBTMA][PF_6_] particles are irregular, with a size distribution of 2~5 μm, and PANI particles are also irregular but they seem to be composed of smaller primary particles. The morphology and size of P[VBTMA][PF_6_]-c-PANI particles are obviously different from PANI particles, but they are very similar to those of P[VBTMA][PF_6_] particles (see Figure 1E). At the same time, no smaller PANI particles appear on the surface of P[VBTMA][PF_6_]-c-PANI particles. According to conductivity measurement, before dedoping by ammonia, the pure PANI particles exhibit high conductivity, while P[VBTMA][PF_6_]-c-PANI particles are insulating, like pure P[VBTMA][PF_6_]. These indicate that PANI particles are capsulated by P[VBTMA][PF_6_]. In addition, it is noted that the morphology and size of P[VBTMA][PF_6_]-c-PANI particles are almost independent of PANI content when the PANI content does not exceed 40 wt %. When the PANI content exceeds 40 wt %, however, PANI particles cannot be fully capsulated by P[VBTMA][PF_6_] and some separated, smaller PANI particles appear. When the PANI content exceeds 50%, we even cannot obtain a good sample because P[VBTMA]Cl cannot be well dissolved in a high concentration of aniline aqueous solution.

#### 3.2.2. FTIR Analysis

**Figure 2:**
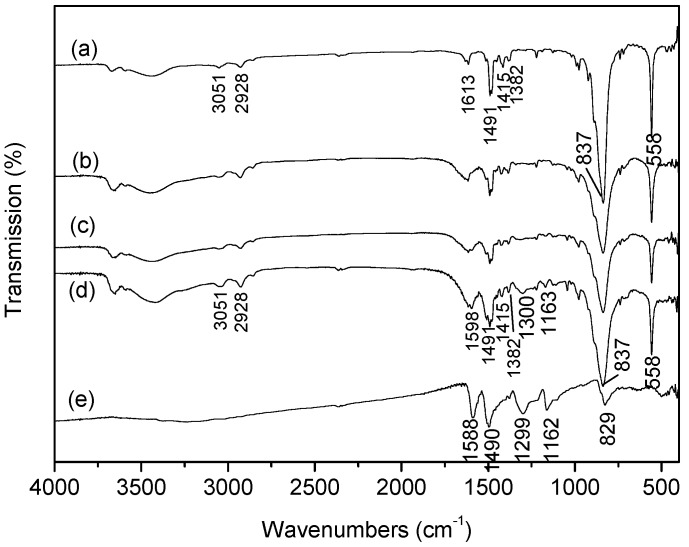
Fourier transform-infrared spectrum (FT-IR) spectra of samples: (**a**) P[VBTMA][PF_6_]; (**b**) P[VBTMA][PF_6_]-c-PANI (10 wt %); (**c**) P[VBTMA][PF_6_]-c-PANI (20 wt %); (**d**) P[VBTMA][PF_6_]-c-PANI (40 wt %); (**e**) PANI.

Figure 2 is the FTIR spectra of P[VBTMA][PF_6_], P[VBTMA][PF_6_]-c-PANI, and PANI particles. P[VBTMA][PF_6_] particles show the characteristic bands corresponding to the poly[VBTMA] cation part at: 3051 cm^−1^ (CH_3_ stretching vibration), 2928 cm^−1^ (CH_2_ stretching vibration), 1613 cm^−1^ and 1491 cm^−1^ (C=C stretching vibration in benzene ring), 1415 cm^−1^ (C–H bending vibration in benzene ring), 1382 cm^−1^ (C–H bending vibration in CH_3_); and the characteristic bands corresponding to the [PF_6_] anion part at: 837 cm^−1^ and 558 cm^−1^ (P–F stretching vibration) (see Figure 2a). PANI particles show the characteristic bands at 1588 cm^−1^ (C=C stretching of quinoid), 1490 cm^−1^ (C=C stretching of benzenoid), 1299 cm^−1^ (C–N stretching of the secondary aromatic amine), 1162 cm^−1^ (electronic-like absorption of N=Q=N (Q representing the quinoid ring)), and 829 cm^−^^1^ (aromatic C–H out-of-plane deformation vibration in linear PANI backbone) (see Figure 2e). P[VBTMA][PF_6_]-c-PANI particles show not only the characteristic bands of P[VBTMA][PF_6_] at 3054 cm^−1^ (CH_3_ stretching vibration), 2928 cm^−1^ (CH_2_ stretching vibration), 837 cm^−1^, and 558 cm^−1^ (P–F stretching vibration), but also the characteristic bands of PANI at 1300 cm^−1^ (C–N stretching vibration) and 1163 cm^−1^ (the electronic-like absorption of N=Q=N). The peaks at 1598 cm^−1^ and 1491 cm^−1^ due to C=C stretching vibration in the benzene ring of P[VBTMA][PF_6_] overlap with the peaks at 1590 cm^−1^ (C=C stretching vibration in quinoid structures) and 1491 cm^−1^ (C=C stretching vibration in benzenoid structures) of PANI. As the PANI content increases, the intensity of bands corresponding to PANI become stronger (see Figure 2b–d). These indicate the coexistence of PANI and P[VBTMA][PF_6_] in the composite particles. However, P[VBTMA][PF_6_] and PANI should not be composited at the molecular scale, because P[VBTMA][Cl] is water soluble, but PANI in particle form is not water soluble before ion exchange. Thus, combining the morphology results with the FT-IR results can confirm that the P[VBTMA][PF_6_]-capsulated PANI composite particles have been obtained by the ion exchange procedure in the aqueous dispersion containing PANI particles.

#### 3.2.3. TG Analysis

**Figure 3:**
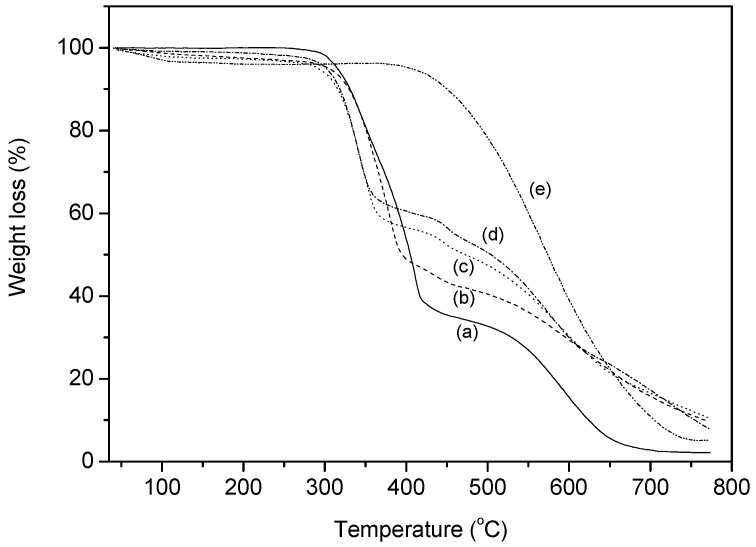
Thermogravimetric analyzer (TGA) curves of samples: (**a**) P[VBTMA][PF_6_]; (**b**) P[VBTMA][PF_6_]-c-PANI (10 wt %); (**c**) P[VBTMA][PF_6_]-c-PANI (20 wt %); (**d**) P[VBTMA][PF_6_]-c-PANI (40 wt %); (**e**) PANI.

Figure 3 shows the TGA curves of P[VBTMA][PF_6_], P[VBTMA][PF_6_]-c-PANI, and PANI particles in air atmosphere. P[VBTMA][PF_6_] particles show a two-step weight loss. The first one can be attributed to the thermal degradation of the polymeric backbone with an onset temperature of 300 °C, and the second one can be attributed to the thermal degradation of hexafluorophosphate counterions with an onset temperature at 500 °C [26]. PANI particles show a one-step weight loss with an onset temperature of around 400 °C, corresponding to the thermal degradation of the polymeric backbone. P[VBTMA][PF_6_]-c-PANI particles show a three-step weight loss, with an onset temperature of 290, 420 and 500 °C, which can respectively be attributed to the thermal degradation of P[VBTMA][PF_6_] and PANI. In particular it is found that, compared with pure PANI and P[VBTMA][PF_6_] particles, the onset temperature of PANI degradation slightly increases, but the onset temperature of P[VBTMA][PF_6_] degradation slightly decreases in P[VBTMA][PF_6_]-c-PANI particles. This is because the P[VBTMA][PF_6_] shell is providing the PANI core with a protective effect.

#### 3.2.4. Zeta Potentials Analysis

Table 1 shows the Zeta potentials of P[VBTMA][PF_6_], P[VBTMA][PF_6_]-c-PANI, and PANI particles. Here, due to no relative material parameter of silicone oil, we used ethanol as the medium to measure the Zeta potentials. In addition, the particles can be dispersed in ethanol better than other solvents and, thus, it can provide a high measurement precision. The Zeta potential of P[VBTMA][PF_6_] particles prepared by post ion-exchange procedure is ~+134 mV, which indicates a positive charged nature. The positive charges may be attributed to the fact that most anions are outside during the post ion-exchange procedure, and it is easy for them to adsorb other cations. The Zeta potential of PANI particles is significantly smaller (~+39 mV). Compared with P[VBTMA][PF_6_] particles, the Zeta potential of P[VBTMA][PF_6_]-c-PANI particles is decreased to ~+110 mV, which indicates that wrapping intrinsically conducting PANI can partly suppress the surface charged state of P[VBTMA][PF_6_] particles. When compared with PANI particles, the higher surface charge for P[VBTMA][PF_6_]-c-PANI particles indicates that the PANI particles are inside P[VBTMA][PF_6_]. 

### 3.3. Electrorheological Property

**Figure 4:**
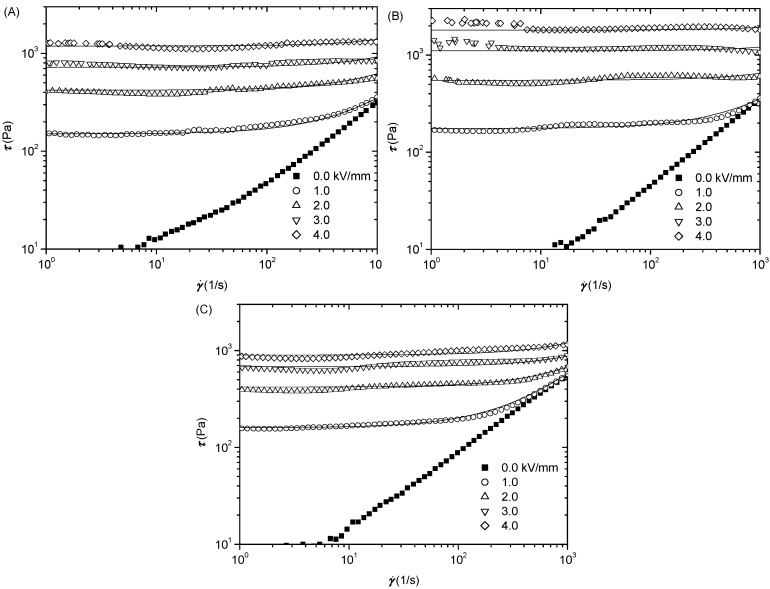
Flow curves of shear stress vs. shear rate for the electrorheological (ER) suspensions: (**A**) P[VBTMA][PF_6_]; (**B**) P[VBTMA][PF_6_]-c-PANI (40 wt %); (**C**) PANI (*φ* = 27 vol %, *T* = 23 °C).

Under a DC electric field, the electroresponsive ER effect of suspensions of P[VBTMA][PF_6_], P[VBTMA][PF_6_]-c-PANI, and PANI particles in silicone oil is measured by a stress-controlled rheometer with a parallel plate system. Figure 4 shows the flow curves of shear stress as a function of shear rate for suspensions at different electric fields. It is seen that without electric fields, these suspensions exhibit a low viscous state. The off-field viscosity of the suspension of P[VBTMA][PF_6_]-c-PANI particles is ~0.35 Pa·s (at 1000 s^−1^), which is very close to the ~0.31 Pa·s of the suspension of P[VBTMA][PF_6_] particles, but lower than the ~0.54 Pa·s of the suspension of PANI particles. The composite particles have a similar particle size and morphology than P[VBTMA][PF_6_] particles, while the PANI particles are composed of smaller primary particles. After the electric field is applied, three suspensions exhibit an obvious increase in shear stress or viscosity and behave like a Bingham plastic material, with large yield stress at various electric field strengths. This is due to the polarization of the dispersed particles and the formation of chain or column-like ER structures under electric fields [27]. Interestingly, it can be seen that the suspension of P[VBTMA][PF_6_]-c-PANI particles shows much larger shear stress and yield stress compared with the suspensions of P[VBTMA][PF_6_] particles and PANI particles, in particular at high electric field strength. At 4 kV/mm of electric field, the yield stress of the suspension of PIL-c-PANI composite particles in silicone oil is about 2.3 kPa, which is twice as high as the 1.2 kPa stress of the suspension of PIL particles and 2.5 times as high as 0.9 kPa stress of the suspension of PANI particles. This indicates that the PIL-c-PANI particles have a distinct enhancement in ER effect.

**Figure 5:**
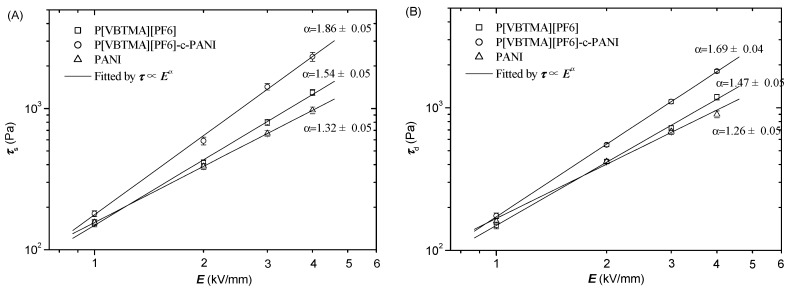
Static yield stress (**A**) and dynamic yield stress (**B**) as a function of electric field strength for ER suspensions: P[VBTMA][PF_6_], P[VBTMA][PF_6_]-c-PANI (40 wt %), and PANI (*φ* = 27 vol %, *T* = 23 °C).

Figure 5 plots the static yield stress ($\tau_{s}$) and dynamic yield stress ($\tau_{d}$) as a function of the electric field strength. The static yield stress corresponds to the stress that makes ER suspensions start to flow, and it characterizes the strength of the suspensions at the yield point, while the dynamic yield stress is defined as the stress making ER suspensions continuously flow, and it characterizes the strength of suspensions in the flow regime [28]. As seen from Figure 5, both static yield stress and dynamic yield stress of the suspension of composite particles are much higher than those of the suspensions of P[VBTMA][PF_6_] and PANI particles, which indicates that the suspension of P[VBTMA][PF_6_]-c-PANI particles has a high strength or enhanced ER effect not only at the yield point, but also in the flow regime. In addition, the correlation of yield stress and electric field strength can be fitted by the power law relation $\tau \propto E^{\alpha }$. Compared with the suspensions of PANI and P[VBTMA][PF_6_] particles, the value of α of the suspension of P[VBTMA][PF_6_]-c-PANI particles is higher, which is also indicating that P[VBTMA][PF_6_]-c-PANI particles have a stronger field-responsive dependence.

**Figure 6:**
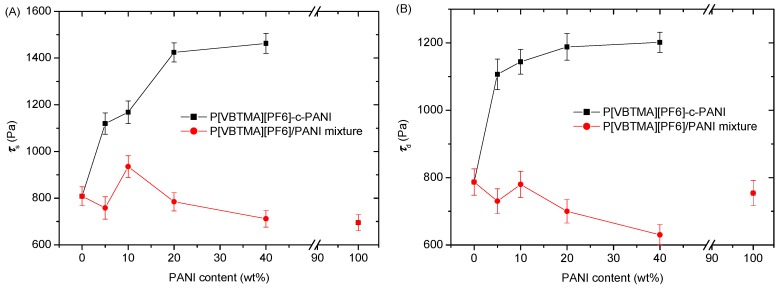
Static yield stress (**A**) and dynamic yield stress (**B**) as a function of PANI content for the suspensions of P[VBTMA][PF_6_]-c-PANI and the corresponding P[VBTMA][PF_6_]/PANI mixture at 3.0 kV/mm of electric field (*φ* = 27 vol %, *T* = 23 °C).

To clarify this enhanced ER effect more clearly, we further compare the ER effect of P[VBTMA][PF_6_]-c-PANI particles with that of a simple mixture of PANI and P[VBTMA][PF_6_] particles at the same PANI content. Figure 6 presents the yield stress as a function of PANI content at 3.0 kV/mm electric field strength. Note that no data over 40% PANI content is presented; we cannot obtain a good sample because P[VBTMA]Cl cannot be well dissolved in high concentration in an aniline aqueous solution. Obviously, both static yield stress and dynamic yield stress of the suspension of P[VBTMA][PF_6_]-c-PANI particles are much stronger than those of corresponding suspension in the P[VBTMA][PF_6_]/PANI mixture. As seen, as the PANI content increases, the yield stresses of the suspension of P[VBTMA][PF_6_]-c-PANI particles increases and becomes saturated, while those of the suspension of the mixture slightly decrease. This clearly indicates that the P[VBTMA][PF_6_]-c-PANI particles do have an enhanced ER effect. Why does this enhancement occur? To clarify the reason, we conduct an investigation of dielectric properties and aligned ER structures below.

### 3.4. Dielectric Properties

We firstly measure the dielectric properties of suspensions, because it has been accepted that ER effect is related to the interfacial polarization of particles in suspensions, and the dielectric properties play an important role in ER effect [29]. Table 2 generalizes the dielectric characteristic of suspensions obtained by the Cole-Cole function fit [22]. It is seen that all suspensions show a dielectric relaxation that can be attributed to the interfacial polarization of dispersed particles in suspension, because the dielectric constant and loss factor of used silicone oil are almost independent of frequency within the measured frequency range. The relaxation time of the suspension of P[VBTMA][PF_6_] particles is the fastest, and that of the suspension of PANI particles is the slowest. However, they are still in the same order, and the difference is small, because the conductivity of P[VBTMA][PF_6_] particles is only slightly higher than that of PANI particles. The relaxation time of the suspension of P[VBTMA][PF_6_]-c-PANI particles is almost same as that of the suspension of P[VBTMA][PF_6_] particles. This may be because the dielectric relaxation of P[VBTMA][PF_6_]-c-PANI particles is dominated by the P[VBTMA][PF_6_] shell, which has a faster relaxation time. The relaxation time of the suspension of P[VBTMA][PF_6_]/PANI mixture is faster than that of the suspension of PANI particles, but it is slower than that of the suspension of P[VBTMA][PF_6_] particles. Meanwhile, the scattering degree of the relaxation time of the suspension of P[VBTMA][PF_6_]/PANI mixture is also broad. This is because both P[VBTMA][PF_6_] and PANI particles are simultaneously dominating the dielectric relaxation process of the suspension of the P[VBTMA][PF_6_]/PANI mixture, but the difference in their relaxation times is small. As a result, the overlapping of two relaxation times results in the broadened scattering degree. However, it is worthy to note that the relaxation time of the suspension of P[VBTMA][PF_6_]/PANI mixture is only slightly slower than that of the suspension of P[VBTMA][PF_6_]-c-PANI particles, and the relaxation strength of the former is also slightly smaller than that of the latter. Thus, although it has been proposed that a good ER effect requires that the ER suspension should first have a faster dielectric relaxation time within an appropriate frequency range of 10^2^–10^5^ Hz, and then have a large dielectric relaxation strength, the differences in the dielectric properties of these samples, in particular for the [VBTMA][PF_6_]/PANI mixture and P[VBTMA][PF_6_]-c-PANI, are so small that they may not result in a large change in ER effect. Therefore, we consider that the small differences in dielectric properties should not be the main reason for the significantly enhanced ER effect of P[VBTMA][PF_6_]-c-PANI particles, and that some other reasons may play a more dominant role.

### 3.5. Microscopic ER Structures

**Figure 7:**
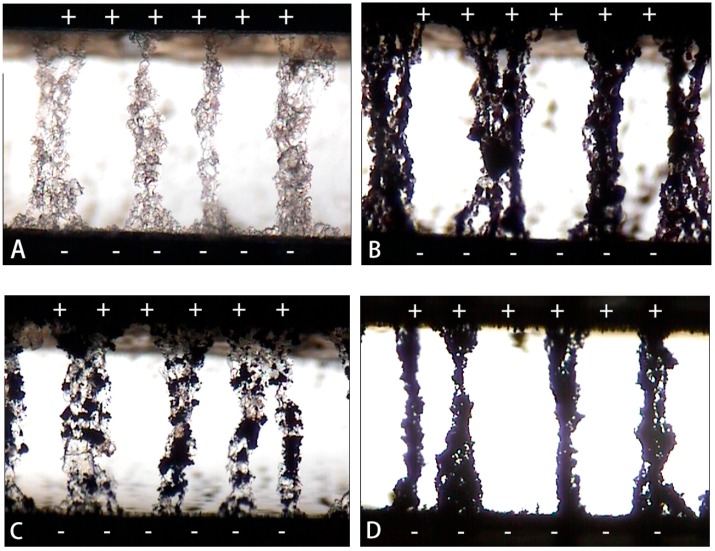
Optical microscopy image of the suspensions under electric fields: (**A**) P[VBTMA][PF_6_]; (**B**) P[VBTMA][PF_6_]-c-PANI (40 wt %); (**C**) P[VBTMA][PF_6_]/PANI (40 wt %) mixture; (**D**) PANI.

Besides the dielectric property, knowing the microscopic ER structure under electric fields is also essential in order to understand ER effect [30,31]. By optical microscopy, we observed the ER structure of diluted suspensions of the above mentioned particles under electric fields. As shown in Figure 7, all the particles can form a column-like ER structure spanning electrodes due to the interparticle attraction of polarized particles under electric fields, but there are differences in the column structures among pure P[VBTMA][PF_6_], P[VBTMA][PF_6_]-c-PANI, P[VBTMA][PF_6_]/PANI mixture and pure PANI particles. From Figure 7A,D, it is seen that the columns formed by P[VBTMA][PF_6_] and PANI particles seem to be not uniform. For P[VBTMA][PF_6_] particles, more particles tend to be attracted or aggregated towards the cathode side and, thus, the columns near the anode side are thinner than those near the cathode side. This may be because PIL particles prepared by post ion-exchange procedure are more easily surface positively charged, according to the Zeta potential analysis. For PANI particles, the particles tend to be attracted toward two electrodes and the central part of the columns seem to be the thinnest. In contrary, the columns formed by P[VBTMA][PF_6_]-c-PANI particles seem to become uniform, and no obvious weak point can be observed. This may be because wrapping PANI into PIL particles has partly suppressed the positively charged surface state, according to the Zeta potential analysis. The columns formed by P[VBTMA][PF_6_]/PANI mixture particles are also uniform, but more transparent P[VBTMA][PF_6_] particles tend to be attracted towards the cathode side, and more black PANI particles tend to be attracted towards the anode side. Based on these observations, we can preliminarily conclude that the formation of P[VBTMA][PF_6_]-c-PANI particles might have to some extent improved the uniformity of the column ER structure and decreased thin column regions. It is known that the shear stress of ER suspension is always dominated by the weakest point of the ER structure. Therefore, the enhanced ER effect of P[VBTMA][PF_6_]-c-PANI particles probably arises from the improvement of the ER structure. In order to clarify this more precisely, we further conduct an oscillation rheological measurement for different samples by the controlled shear stress mode.

### 3.6. Oscillatory Study

**Figure 8:**
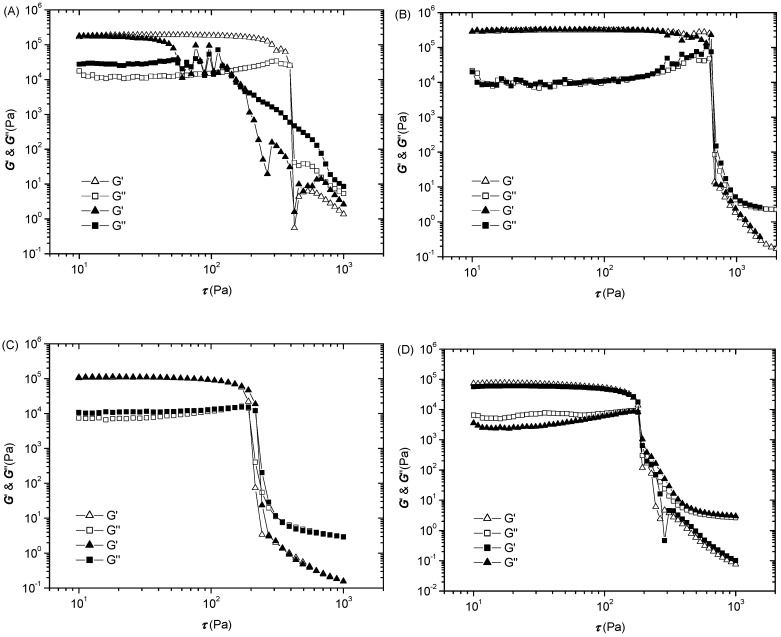
Storage modulus and loss modulus as a function of stress of the suspensions at 2.0 kV/mm electric field: (**A**) P[VBTMA][PF_6_]; (**B**) P[VBTMA][PF_6_]-c-PANI; (**C**) P[VBTMA][PF_6_]/PANI mixture; (**D**) PANI. Solid points were obtained when the rotation side of the electrode was anode, and open points were obtained when the rotation side of the electrode was cathode (*φ* = 27 vol %, *T* = 23 °C).

Since the ER suspension usually shows a solid-like state due to the formation of the ER structure under the electric field, the viscoelastic moduli as a function of applied shear stress presents an initial plateau corresponding to the linear viscoelastic behavior. Meanwhile, the storage modulus is always higher than the loss modulus, and the ER suspension has a dominant elastic response. As the stress amplitude increases and exceeds a critical value, the viscoelastic moduli exhibit a sharp decrease that represents the rapture of the ER structure and the beginning of flow. The characteristic stress as this change happens is named the critical stress ($\tau_{c}$), which is very sensitive to the weakest point in the ER structure [30]. Therefore, we can precisely understand the ER structure by the evolution of the viscoelastic moduli with the amplitude of the oscillatory stress. Figure 8 shows storage modulus (*G*′) and loss modulus (*G*″) as a function of stress amplitude at the frequency of 1 Hz and at 2.0 kV/mm electric field strength for the suspensions of pure P[VBTMA][PF_6_], P[VBTMA][PF_6_]-c-PANI and P[VBTMA][PF_6_]/PANI mixture, and pure PANI particles. Here, having considered the distribution of shear deformation rate and to clarify the influence of the ER structure on properties, we carried out a comparable measurement by exchange the polarity of the rotation side of the electrode. As shown in Figure 8, under the electric field, the suspensions do show a solid-like state according to the fact that *G*′ is larger than *G*″, and *G*′ remains constant before the stress exceeds the yield value, which is called linear viscoelastic behavior. As the stress amplitude increases and exceeds a critical value, the viscoelastic moduli exhibit a sharp decrease and the suspensions start to flow. However, there are significant differences in the amplitude of the storage modulus and the critical stress for different samples.

The first difference concerns the amplitude of the storage modulus. It can be seen that, compared with the suspensions of P[VBTMA][PF_6_], the P[VBTMA][PF_6_]/PANI mixture, and PANI particles, the suspension of P[VBTMA][PF_6_]-c-PANI particles has the largest storage modulus. This indicates that the ER structure or solidification level of the suspension of P[VBTMA][PF_6_]-c-PANI particles is the strongest, which also supports its enhanced ER effect. The storage modulus of the suspension of PANI is smaller than that of the suspension of P[VBTMA][PF_6_] particles, which is also in accordance with its thinner column-like ER structure and ER effect. The storage modulus of the suspension of the P[VBTMA][PF_6_]/PANI mixture is very similar to that of the suspension of the PANI particles. This hints that the storage modulus may be mainly dominated by the weaker aggregation region of PANI particles in columns rather than the aggregation region of P[VBTMA][PF_6_] particles.

**Figure 9:**
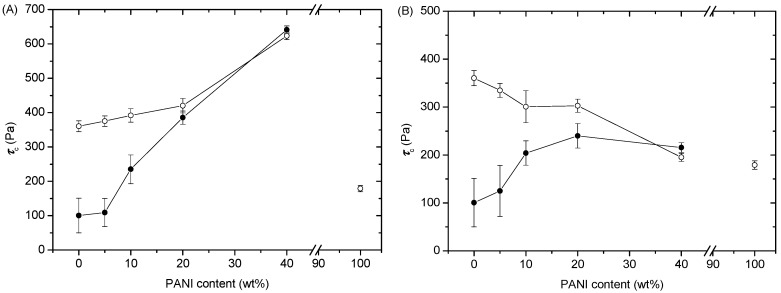
Critical stress as a function of PANI content for the suspensions of P[VBTMA][PF_6_]-c-PANI (**A**) and P[VBTMA][PF_6_]/PANI mixture; (**B**) at 2.0 kV/mm electric field. Solid points were obtained when the rotation side of the electrode was anode, and open points were obtained when the rotation side of the electrode was cathode (*φ* = 27 vol %, *T* = 23 °C).

The second difference concerns the critical stress at transformation point from a solid-like state to flow state. From Figure 8, it can be seen that the suspension of P[VBTMA][PF_6_]-c-PANI particles has the largest critical stress compared to the suspensions of P[VBTMA][PF_6_], PANI, and the P[VBTMA][PF_6_]/PANI mixture. This further supports that the ER structure or solidification level of the suspension of P[VBTMA][PF_6_]-c-PANI particles is the strongest. However, the most interesting aspect is the difference in the amplitude of critical stresses obtained in the case of the cathode as the rotation side, and in the case of the anode as the rotation side, for different samples. To show this difference, the values of $\tau_{c}$ as a function of PANI content are plotted in Figure 9. It is seen that, for the suspension of P[VBTMA][PF_6_] particles, the value of critical stress is smaller when the rotation side of the electrode is anode, compared with when the rotation side of the electrode is cathode. According to the optical microscopic observations before, we consider this should be related to the fact that the thinnest column region or the weakest point in the columns formed by P[VBTMA][PF_6_] particles is located near the anode side. As a result, the rapture of the columns along the surface of the electrode easily occurs when the rotation side of the electrode is anode, because the value of the shear rate is at its maximum at the surface of the rotation side. For the suspension of PANI particles, the value of critical stress obtained in the case of cathode as rotation side is almost same with that obtained in the case of anode as rotation side. This is also in accordance with the fact that PANI particles tend to be attracted toward two electrodes, and the thinnest column region or the weakest point is located in the central part of the columns. For the suspension of P[VBTMA][PF_6_]-c-PANI particles, the difference in the amplitude of critical stresses obtained in the case of the cathode as rotation side, and in the case of anode as rotation side, gradually becomes smaller with the increase of PANI content. When the PANI content is close to 40 wt %, the value of critical stress obtained in the case of cathode as rotation side is very close to that obtained in the case of anode as rotation side. The similar phenomenon is also observed for the suspension of P[VBTMA][PF_6_]/PANI mixture. According to the optical microscopic observation, we can also consider this is related to the fact that the columns formed by particles become more and more uniform with the addition of PANI. However, it can also be noted that both the critical stress obtained in the case of cathode as rotation side, and the critical stress obtained in the case of anode as rotation side, increase with the increase of PANI content for the suspension of P[VBTMA][PF_6_]-c-PANI particles. Meanwhile, the critical stress obtained in the case of cathode as rotation side decreases with the increase of PANI content in the suspension of P[VBTMA][PF_6_]/PANI mixture. This indicates that, although the simple mixing of PANI particles into the suspension of P[VBTMA][PF_6_] particles can improve the uniformity of column structure, the strength of columns formed by P[VBTMA][PF_6_]-c-PANI particles is much stronger compared with the strength of columns formed by P[VBTMA][PF_6_]/PANI mixture. This can be explained by the fact that the PANI particles cannot directly contact in columns formed by P[VBTMA][PF_6_]-c-PANI particles, while the PANI particles are easy to directly contact in columns formed by P[VBTMA][PF_6_]/PANI mixture particles. As a result, the weaker interaction between PANI particles dominates the strength of the suspension of the P[VBTMA][PF_6_]/PANI mixture as the PANI content increases. Thus, the critical stress result has also revealed that the improvement in the columns formed by P[VBTMA][PF_6_]-c-PANI particles may be responsible for the enhanced ER effect. Since there are not significant differences in the dielectric properties, we consider that the improvement of the column-like ER structure may be associated with the fact that wrapping PANI into P[VBTMA][PF_6_] particles has partly suppressed the positively charged surface state.

## 4. Conclusions

By using intrinsically conducting PANI as a core, we prepared a type of PILs-capsulated PANI composite particles in order to both overcome the surface charged character of pure PILs particles prepared by post ion-exchange procedure and enhance ER effect. Under electric fields, the PILs-capsulated PANI particles were found to show an enhanced ER effect when compared with that of pure PIL particles, PANI particles, and their simple mixture. By using dielectric spectroscopy, microscopic observation, and oscillation rheology, we studied the origin of enhanced ER effect. The results indicated that wrapping PANI into PILs could partly suppress the positively charged state of PILs particles prepared by post ion-exchange procedure and improve the ER structure. This suppression should be responsible for the enhanced ER effect of PILs-capsulated PANI composite particles. 

## Figures and Tables

**Table 1:** Zeta potentials of P[VBTMA][PF_6_], P[VBTMA][PF_6_]-c-PANI, and PANI particles.

Sample	P[VBTMA][PF_6_]	P[VBTMA][PF_6_]-c-PANI	PANI
Zeta potentials (mV)	+134	+110	+39

**Table 2:** Dielectric characteristics of the suspensions of P[VBTMA][PF_6_], PANI, P[VBTMA][PF_6_]-c-PANI (40 wt %), and P[VBTMA][PF_6_]/PANI (40 wt %) mixture (*φ* = 15 vol %, *T* = 23 °C).

Sample	*ε_0_* ^(a)^	*ε_∞_* ^(b)^	*∆ε* ^(c)^	*τ*(s) ^(d)^	*α* ^(e)^	*σ_p_* (S/cm) ^(f)^
P[VBTMA][PF_6_]	4.31	2.91	1.40	0.0022	1.26	~1.0 × 10^−9^
PANI	4.28	2.68	1.60	0.0040	1.20	~7.5 × 10^−10^
P[VBTMA][PF_6_]-c-PANI	4.35	2.65	1.70	0.0023	1.24	~2.6 × 10^−10^
P[VBTMA][PF_6_]/PANI	4.48	2.8	1.68	0.0030	1.12	-

^(a)^ The limit values of the dielectric constant at the frequencies below the relaxation frequency; ^(b)^ The limit values of the dielectric constant at the frequencies above the relaxation frequency; ^(c)^ The dielectric constant increment or relaxation strength denoted by *∆ε = ε_0_ − ε_∞_*; ^(d)^ The dielectric relaxation time denoted by *τ* = 1/(2π*f*_max_), where *f*_max_ the relaxation frequency defined by a local maximum of the dielectric loss factor *ε″*; ^(e)^ The scattering degree of relaxation time *τ*; ^(f)^ The conductivity of particles calculated by Equation *σ* = *φσ_p_* + (1 − *φ*)*σ_f_*.

## References

[1] Capadona J.R., Shanmuganathan K., Tyler D.J., Rowan S.J., Weder C. (2008). Stimuli-responsive polymer nanocomposites inspired by the sea cucumber dermis. Science.

[2] Lampert C.M. (2004). Chromogenic smart materials. Mater. Today.

[3] Halsey T.C. (1992). Electrorheological fluids. Science.

[4] Liu Y.D., Choi H.J. (2012). Electrorheological fluids: Smart soft matter and characteristics. Soft Matter.

[5] Coulter J.P., Weiss K.D., Carlson J.D. (1993). Engineering applications of electrorheological materials. J. Intell. Mater. Syst. Struct..

[6] Sheng P., Wen W. (2012). Electrorheological fluids: Mechanisms, dynamics, and microfluidics applications. Annu. Rev. Fluid Mech..

[7] Tao R., Tang H., Tawhid-Al-Islam K., Du E., Kim J. (2016). Electrorheology leads to healthier and tastier chocolate. Proc. Natl. Acad. Sci. USA.

[8] Winslow W.M. (1949). Induced fibration of suspensions. J. Appl. Phys..

[9] Hao T. (2001). Electrorheological fluids. Adv. Mater..

[10] Wen W., Huang X., Yang S., Lu K., Sheng P. (2003). The giant electrorheological effect in suspensions of nanoparticles. Nat. Mater..

[11] Lu K., Shen R., Wang X., Sun G., Wen W. (2005). The electrorheological fluids with high shear stress. Int. J. Mod. Phys. B.

[12] See H. (1999). Advances in modelling the mechanisms and rheology of electrorheological fluids. Korea-Aust. Rheol. J..

[13] Weiss K.D., Carlson J.D., Coulter J.P. (1993). Review: Material aspects of electrorheological systems. J. Intell. Mater. Syst. Struct..

[14] Yin J.B., Zhao X.P. (2002). Preparation and electrorheological activity of mesoporous rare-earth-doped TiO_2_. Chem. Mater..

[15] Lee Y.H., Kim C.A., Jang W.H., Choi H.J., Jhon M.S. (2001). Synthesis and electrorheological characteristics of microencapsulated polyaniline particles with melamine–formaldehyde resins. Polymer.

[16] Cao Y., Choi H.J., Zhang W.L., Wang B., Hao C., Liu J. (2016). Eco-friendly mass production of poly(*p*-phenylenediamine)/graphene oxide nanoplatelet composites and their electrorheological characteristics. Compos. Sci. Technol..

[17] Wang B.X., Liu C.J., Yin Y.C., Yu S., Chen K., Liu P., Liang B. (2013). Double template assisting synthesized core–shell structured titania/polyaniline nanocomposite and its smart electrorheological response. Compos. Sci. Technol..

[18] Quadrat O., Stejskal J. (2006). Polyaniline in electrorheology. J. Ind. Eng. Chem..

[19] Bloodworth R., Wendt E. (1996). Materials for ER fluids. Int. J. Mod. Phys. B.

[20] Dong Y.Z., Yin J.B., Zhao X.P. (2014). Microwave-synthesized poly(ionic liquid) particles: A new material with high electrorheological activity. J. Mater. Chem. A.

[21] Mecerreyes D. (2011). Polymeric ionic liquids: Broadening the properties and applications of polyelectrolytes. Prog. Polym. Sci..

[22] Dong Y.Z., Yin J.B., Yuan J.H., Zhao X.P. (2016). Microwave-assisted synthesis and high-performance anhydrous electrorheological characteristic of monodisperse poly (ionic liquid) particles with different size of cation/anion parts. Polymer.

[23] Yuan J.Y., Mecerreyes D., Antonietti M. (2013). Poly(ionic liquid)s: An update. Prog. Polym. Sci..

[24] Men Y.J., Kuzmicz D., Yuan J.Y. (2014). Poly (ionic liquid) colloidal particles. Curr. Opin. Colloid Interface Sci..

[25] Marcilla R., Ochoteco E., Pozo-Gonzalo C., Grande H., Pomposo J.A., Mecerreyes D. (2005). New Organic Dispersions of Conducting Polymers Using Polymeric Ionic Liquids as Stabilizers. Macromol. Rapid Commun..

[26] Yu G.R., Li Q.Z., Li N., Man Z.W., Pu C.H., Asumana C., Chen X.C. (2014). Synthesis of new crosslinked porous ammonium-based poly (ionic liquid) and application in CO_2_ adsorption. Polym. Eng. Sci..

[27] Block H., Kelly J.P. (1988). Electro-rheology. J. Phys. D Appl. Phys..

[28] Block H., Rattray P., Havelka K.O., Filisko F.E. (1995). Recent developments in ER fluids. Progress in Electrorheology.

[29] Ikazaki F., Kawai A., Uchida K., Kawakami T., Edamura K., Sakurai K., Anzai H., Asako Y. (1998). Mechanisms of electrorheology: The effect of the dielectric property. J. Phys. D Appl. Phys..

[30] Hanaoka R., Hotta K., Anzai H., Sakurai K., Kuroda S. (2000). Internal structure and ER properties in ER suspensions of disperse system under DC electric field. Electr. Eng. Jpn..

[31] Chin B.D., Park O.O. (2000). Rheology and microstructures of electrorheological fluids containing both dispersed particles and liquid drops in a continuous phase. J. Rheol..

